# Diagnosis of 5α–reductase 2 deficiency: is measurement of dihydrotestosterone essential?

**DOI:** 10.1186/1687-9856-2013-S1-P186

**Published:** 2013-10-03

**Authors:** Angel OK Chan, WM But, CY Lee, YY Lam, KL Ng, Joanna Tung, PT Cheung, Doris Chan, WY Tse, CC Shek

**Affiliations:** 1 Department of Pathology, Queen Elizabeth Hospital, Hong Kong; 2 Department of Paediatrics, Queen Elizabeth Hospital, Hong Kong; 3 Department of Paediatrics, Caritas Medical Centre, Hong Kong; 4 Department of Paediatrics, Kwong Wah Hospital, Hong Kong; 5 Department of Paediatrics, United Christian Hospital, Hong Kong; 6 Department of Paediatrics, Queen Mary Hospital, Hong Kong; 7 Department of Medicine, Caritas Medical Centre, Hong Kong

## 

5α-reductase 2 deficiency (5ARD) is a known cause of 46,XY disorders of sex development. Classical biochemical hallmarks include a normal to high male level of serum testosterone, low level of dihydrotestosterone (DHT) and a raised testosterone/DHT ratio at baseline and/or after human chorionic gonadotropin stimulation. However, equivocal results are not uncommonly encountered, potentially misleading the diagnosis and therefore wrong sex assignment. Our objective is to propose laboratory diagnostic algorithms other than measuring DHT for diagnosing 5ARD. A retrospective review was conducted on all our local 5ARD patients with urinary steroid profiling (USP) or *SRD5A2* genetic testing performed. Literature review was also carried out on all the reported 5ARD cases in the past ten years. A total of 16 local 5ARD patients were studied. Fifteen patients were diagnosed by USP, with characteristically low 5α- to 5β-reduced steroid metabolite ratios. Since insignificant amount of 5α- and 5β-reduced steroid metabolites is excreted under three months of age, a neonate had the genetic testing performed directly. Altogether, 12 patients underwent mutational analysis of the *SRD5A2* gene, all had two mutations detected to confirm the diagnosis. Four patients had DHT measured, with one of them reaching the diagnostic cutoff of 5ARD after human chorionic gonadotropin-stimulation. A hundred and forty-three 5ARD patients were reported in 23 publications in the review period. Ninety-five percent of them had two mutations detected to confirm the diagnosis. Less than half of all these patients had DHT tested. With the high mutation detection rate in 5ARD patients, we propose analysing the *SRD5A2* gene in all newborns with 46,XY DSD for an early diagnosis before sex assignment and any surgical intervention. When USP is readily available, it should also be used as a first-line test to guide subsequent blood testing. In conclusion, 5ARD can be confidently diagnosed by mutational analysis of the *SRD5A2* gene and by USP. Testing the DHT level is not essential to the diagnosis of this condition. The role of this hormone test in diagnosing 5ARD has been over-emphasized.

